# Editorial: Six-membered heterocycles: their synthesis and bio applications

**DOI:** 10.3389/fchem.2023.1229825

**Published:** 2023-06-08

**Authors:** Pezhman Shiri, Atefeh Roosta, Sorour Ramezanpour, Ali Mohammad Amani

**Affiliations:** ^1^ Department of Medical Nanotechnology, School of Advanced Medical Sciences and Technologies, Shiraz University of Medical Sciences, Shiraz, Iran; ^2^ Pharmaceutical Sciences Research Center, Shiraz University of Medical Sciences, Shiraz, Iran; ^3^ Department of Chemistry, Tarbiat Modares University, Tehran, Iran; ^4^ Department of Chemistry, K. N. Toosi University of Technology, Tehran, Iran

**Keywords:** six-membered heterocycles, pharmacological properties, synthesis, natural products, bioapplications, ring structure

**FIGURE 1:**
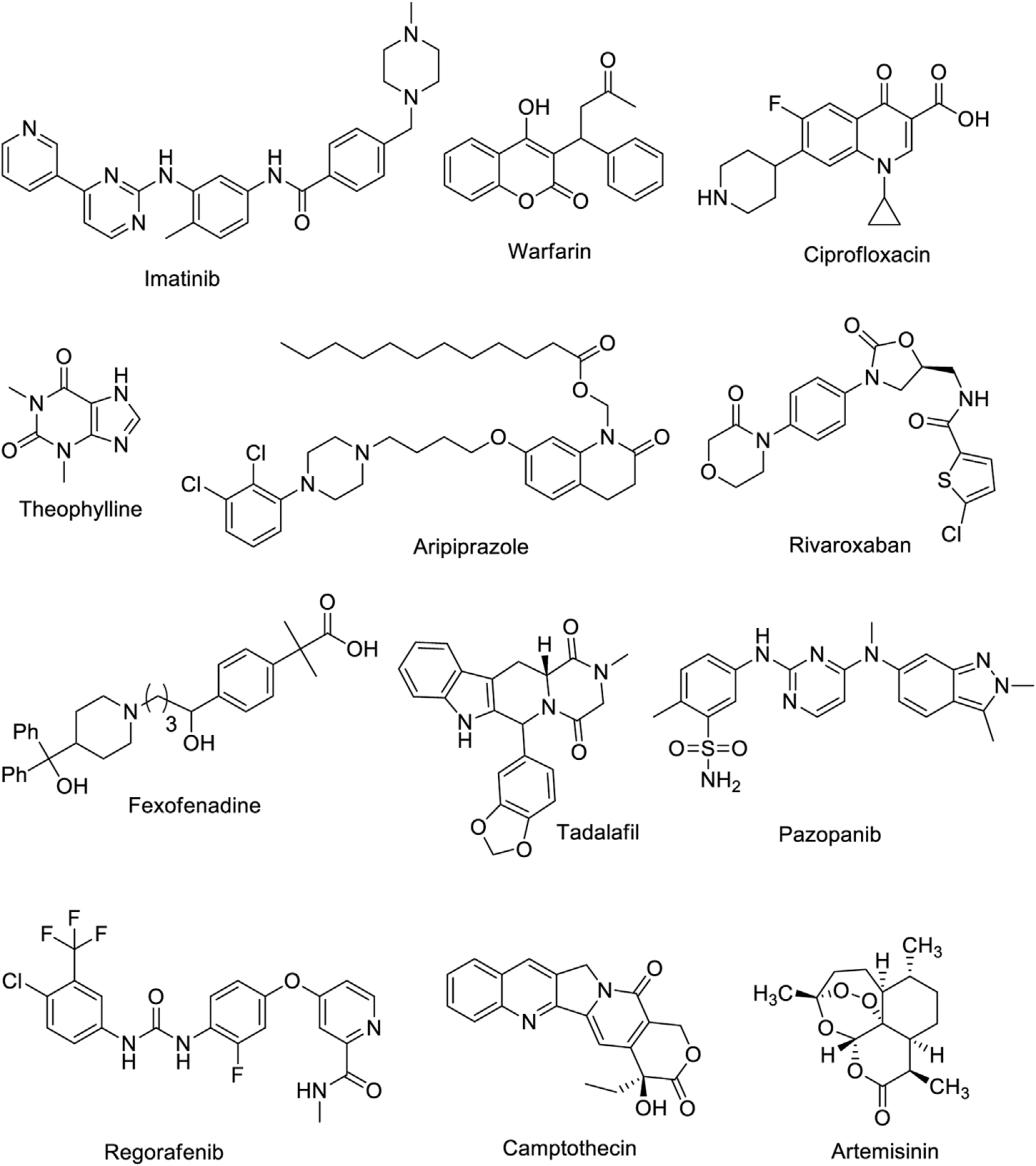
Chemical structure of medicines and natural products containing six-membered heterocycles.

In organic chemistry, six-membered heterocycles refer to molecules that comprise a ring structure with six atoms, and at least one of the atoms is different from carbon. Various applications exist for these heterocyclic compounds both in organic synthesis and pharmaceutical production owing to their unique chemical properties. A variety of methods are available for the synthesis of six-membered heterocycles, including cyclization reactions, cycloaddition reactions, and oxidative coupling reactions (Shiri et al., 2022; Lattanzi, 2023). In terms of bioapplications, six-membered heterocycles have been deeply explored for their pharmacological properties (Cascorbi, 2012; Kadow et al., 2013). Medicines containing six-membered heterocyclic compounds include (Figure 1):

**Imatinib**: a medicine for the treatment of chronic myelogenous leukemia (CML) and gastrointestinal stromal tumors (GIST).

**Warfarin**: a drug prescribed to prevent blood clots.

**Ciprofloxacin**: prescribed for the treatment of bacterial infections.

**Theophylline**: a drug used in the treatment of asthma and other respiratory diseases.

**Aripiprazole**: prescribed for schizophrenia, bipolar disorder, and major depressive disorder.

**Rivaroxaban**: a medication that prevents blood clots and strokes in people with atrial fibrillation or deep vein thrombosis.

**Fexofenadine**: For treating allergy symptoms such as hay fever and hives.

**Tadalafil**: for treating erectile dysfunction and pulmonary arterial hypertension.

**Pazopanib**: a medication used for advanced renal cell carcinoma (kidney cancer) and soft tissue sarcoma, as well as certain types of ovarian cancer.

**Regorafenib:** a treatment for certain types of cancer, including colorectal cancer and gastrointestinal stromal tumors (GIST) that no longer respond to other treatments.

The potential therapeutic applications of natural products containing six-membered heterocycles (Figure 1) have gained significant attention over the past few years. Plants, fungi, and marine organisms contain these compounds. An example of a natural product containing six-membered heterocycles is camptothecin, previously discovered as a potent anticancer agent. Camptothecin contains a six-membered lactone ring and exhibits a unique mechanism of action that targets cancer cells while sparing healthy cells. Artemisinin, which is derived from the sweet wormwood plant and is used to treat malaria, is another natural product that contains six-membered heterocycles. Artemisinin also contains a six-membered lactone ring and exhibits considerable antimalarial activity. The synthesis and bioapplication of six-membered heterocycles from natural products has become an active research field. Researchers are exploring new strategies to synthesize these molecules and identify potential therapeutic compounds. With ongoing attempts to identify potential natural sources for these molecules, potentially more six-membered heterocycle-containing natural products could be discovered in the future, opening up new drug discovery possibilities.

Presented herein is a summary of the research and review articles which have been published in this topic.

Hossaini et al. reported on their study of six-component synthesis and biological activity of novel spiropyridoindolepyrrolidine derivatives. This combined experimental and theoretical investigation showed promising results, with the synthesized compounds exhibiting high antioxidant and antimicrobial abilities. The study highlights the potential of this approach to produce effective new compounds with diverse biological activities.

Chehreghani et al. synthesized a molecular imprinted polymer (MIP) for quetiapine using precipitation polymerization. The MIP showed high capability as a pharmaceutical carrier for quetiapine, with the highest adsorption rates observed at pH 7. The drug release kinetics followed the Higuchi and Korsmeyer-Peppas models under different pH conditions. Moreover, DFT-based quantum chemical descriptors revealed strong interactions between the polymeric host and the quetiapine drug. Quetiapine is an FDA-approved medication for schizophrenia, acute manic episodes, major depressive disorder, and used off-label to manage generalized anxiety disorder and other conditions.

Ezzatzadeh et al. reported on the synthesis and evaluation of new spiro-1,2,4-triazine derivatives for their antioxidant activity, where Ag/Fe_3_O_4_/CdO@MWCNT MNCs were applied as efficient organometallic nanocatalysts.

Mohammadi et al. investigated the carbon fixation of CO_2_ via cyclic reactions with borane in gaseous atmosphere leading to formic acid (and metaboric acid) through a potential energy surface (PES) study.

Tavakol and Firouzi reported on the synthesis of 14*H*-dibenzoxanthenes using Sn(II)/nano silica as an efficient catalyst in green media. The researchers prepared the catalyst by depositing SnCl_2_.2H_2_O onto nano-silica. They then used this catalyst in a one-pot condensation reaction of *β*-naphthol with various aliphatic and aromatic aldehydes to synthesize several xanthene derivatives. The reaction was carried out using ethanol as the solvent, 10 mol percent of the catalyst, at reflux condition for 3 h, resulting in yields ranging from 48% to 94%.

Khanlari et al. studied the application of an oxycodone-templated molecular imprinted polymer for selective adsorption of the drug from human blood plasma in a real biological environment. The researchers employed a joint experimental and density functional theory approach to investigate the efficiency of the polymer in capturing oxycodone from complex biological matrices.

Alshammari et al. conducted a collaborative study involving researchers from various countries, including Egypt, Germany, the United States, and Saudi Arabia. The research aimed to design and synthesize new thiazolidinone/uracil derivatives as antiproliferative agents targeting EGFR and/or BRAFV600E, with the goal of developing novel compounds that could effectively inhibit the growth of cancer cells by targeting specific proteins.

Borah et al. from India published a review article in our Research Topic focusing on recent advances and prospects in the organocatalytic synthesis of quinazolinones.
